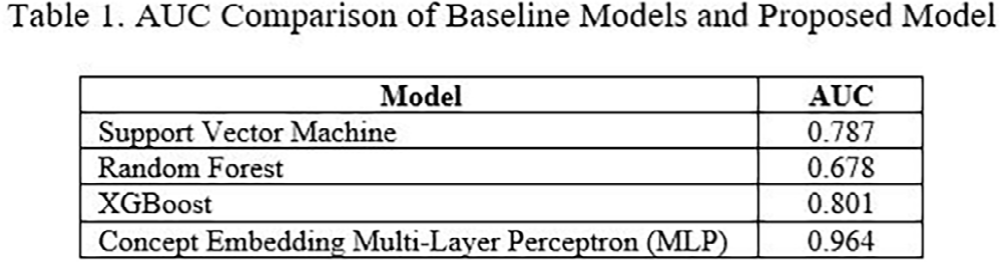# Interpretable AI‐Driven Biomarker Kits for Early Detection of Dementia using Multimodal SCAN Data

**DOI:** 10.1002/alz70856_101296

**Published:** 2025-12-25

**Authors:** Ali Eslamian, Qiang Cheng, Colleen Pappas, Christopher E. Bauer, Brian T Gold

**Affiliations:** ^1^ University of Kentucky, Lexington, KY, USA; ^2^ Sanders‐Brown Center on Aging, Lexington, KY, USA

## Abstract

**Background:**

Establishing standardized multimodal neuroimaging markers of preclinical dementia‐states could improve early detection for clinical trials. The SCAN [1] initiative harmonizes PET, MRI, and cerebrospinal fluid (CSF) biomarkers across multiple Alzheimer's Disease Research Centers (ADRCs) to enable cross‐ADRC site analyses. However, integrating diverse data modalities to predict cognitive impairment remains a challenge. Our study aims to develop an interpretable AI model leveraging SCAN data to predict the Clinical Dementia Rating (CDR) global score, a measure of global cognitive impairment. The CDR evaluates both cognitive and functional abilities. As a first step toward that goal, we tested the accuracy of several AI models in predicting CDR scores from a combination of MRI from the SCAN data repository.

**Method:**

We analyzed SCAN‐compliant summary measures from MRI biomarkers, UDS‐3 assessments from the National Alzheimer's Coordinating Center (NACC), based on data from 2,006 participants (59% women). The imaging metrics included brain volumes, cortical thickness, surface areas. We developed a deep neural network (DNN) model using a multimodal fusion strategy to integrate these diverse data types and predict CDR categories (0 and 0.5). The model employed concept embedding to project heterogeneous input from different modalities into a shared latent space, capturing both structural brain changes and cognitive performance measures. The selected features included over 200 neuroimaging‐derived metrics, such as total brain volumes, hippocampal volumes, and cortical measurements across various regions. Interpretability was achieved using Shapley Additive Explanations (SHAP), which quantified feature importance and explained individual predictions.

**Result:**

Preliminary experiments were conducted using several machine learning models (Table 1) to establish baseline performance for classifying AD versus Cognitively Normal (CN) categories. The Concept Embedding MLP demonstrated superior performance for CDR classifications. SHAP analysis identified key predictors, including hippocampal volumes, anterior cingulate thickness, as important contributors. The model provides both global feature importance patterns and local explanations for individual predictions.

**Conclusion:**

Our AI approach integrates multimodal neuroimaging to predict CDR scores, aiding early Alzheimer's diagnosis. Interpretability highlights key features, and future work will include longitudinal data and external validation.

[1] National Alzheimer's Coordinating Center (NACC). (n.d.). SCAN: NACC Imaging Portal. Retrieved from https://scan.naccdata.org/, accessed 2025.